# Glycosylated Delphinidins, Natural Antioxidants, Functionally Modulate the COX-2/PGE_2_ Axis and Downregulate MDR1 in Glioblastoma Cells

**DOI:** 10.3390/antiox15081005

**Published:** 2026-08-13

**Authors:** Constanza Cuevas, Yessica Nahuelpan, Darling Haro, Pamela Silva, Vicente Villagran, María A. Gleisner, Flavio Salazar-Onfray, Juan P. Muñoz, Christian Pávez, Noemi Garcia-Romero, Angel Ayuso-Sacido, Rody San Martin, Diego Carrillo-Beltrán, Claudia Quezada-Monrás

**Affiliations:** 1 Laboratorio de Oncovirología Molecular, Instituto de Bioquímica y Microbiología, Facultad de Ciencias, Universidad Austral de Chile, Valdivia 5090000, Chile; constanza.cuevas@alumnos.uach.cl (C.C.); yessica.nahuelpan@alumnos.uach.cl (Y.N.); darling.haro@alumnos.uach.cl (D.H.); christian.pavez@alumnos.uach.cl (C.P.); 2 Laboratorio de Biología Tumoral, Instituto de Bioquímica y Microbiología, Facultad de Ciencias, Universidad Austral de Chile, Valdivia 5090000, Chile; pamela.silva01@uach.cl (P.S.); vicente.villagran@alumnos.uach.cl (V.V.); 3 Millennium Institute on Immunology and Immunotherapy, Facultad de Ciencias, Universidad Austral de Chile, Valdivia 5110566, Chile; maria.gleisner@uchile.cl (M.A.G.); fsalazar@uchile.cl (F.S.-O.); 4 Disciplinary Program of Immunology, Institute of Biomedical Sciences, Faculty of Medicine, Universidad de Chile, Santiago 8380453, Chile; 5 Laboratorio de Bioquímica, Departamento de Química, Facultad de Ciencias, Universidad de Tarapacá, Arica 1000007, Chile; jpmunozb@academicos.uta.cl; 6 Faculty of Experimental Sciences, Universidad Francisco de Vitoria, 28223 Madrid, Spain; noemi.garcia@ufv.es (N.G.-R.);; 7 Brain Tumour Laboratory, Fundación Vithas, Grupo Hospitales Vithas, 28223 Madrid, Spain; 8 Preclinical Models of Brain Tumors Lab (BTL), Unidad Funcional de Enfermedades Crónicas (UFIEC), Instituto de Salud Carlos III (ISCIII), 28223 Madrid, Spain; 9 Laboratorio de Patología Molecular, Instituto de Bioquímica Y Microbiología, Universidad Austral de Chile, Valdivia 5090000, Chile; rodysanmartin@uach.cl

**Keywords:** delphinidin-3-glucoside, natural antioxidants, anthocyanins, glioblastoma, MDR1/P-glycoprotein, COX-2, chemoresistance, inflammation, NF-κB

## Abstract

The multidrug resistance (MDR) phenotype, mediated by transporters such as MDR1/P-glycoprotein (P-gp), represents a critical obstacle in the treatment of glioblastoma (GB). Natural antioxidants such as glycosylated delphinidins—anthocyanins abundant in Chilean berries, particularly the native species *Aristotelia chilensis* (maqui)—have emerged as bioactive compounds that modulate inflammation and cancer-related pathways. In this context, the regulation of inflammation-associated signaling pathways, including COX-2 and NF-κB, represents a potential strategy to overcome chemoresistance. In this study, we evaluated the effect of delphinidin-3-glucoside (DEL-3) on the COX-2/PGE_2_ axis, MDR1 expression, and sensitivity to chemotherapeutic agents in U87-MG and GBM38 glioblastoma cells and in an NOD-SCID xenograft model using ELISA, RT-qPCR, Western blot, and luciferase reporter assays. DEL-3 reduced PGE_2_ production, consistent with functional modulation of the COX-2/PGE_2_ pathway, and downregulated MDR1 expression at both transcriptional and protein levels, in agreement with reduced promoter activity. Functionally, combined treatment with DEL-3 and MDR1-substrate chemotherapeutic agents enhanced cytotoxicity in U87-MG cells. Although DEL-3 did not significantly reduce tumor growth in a NOD-SCID xenograft model, MDR1 expression was reduced in treated tumors, indicating in vivo modulation of the target. Overall, these findings suggest that DEL-3, as a natural antioxidant, modulates inflammation-associated mechanisms and downregulates MDR1, thereby potentially enhancing the sensitivity of glioblastoma cells to MDR1-substrate chemotherapeutic agents.

## 1. Introduction

The development of the multidrug resistance (MDR) phenotype is a major obstacle to chemotherapy efficacy, with ATP-binding cassette (ABC) transporters, such as MDR1/P-glycoprotein (P-gp), playing key roles by limiting the intracellular accumulation of antitumor drugs [1]. In this context, strategies to overcome chemoresistance have focused on modulating the activity of these transporters to improve intracellular drug retention and reduce required therapeutic doses [2]. Notably, increasing evidence suggests that inflammation-associated pathways and redox-sensitive signaling mechanisms contribute to the regulation of MDR transporters, linking oxidative stress and cancer therapy resistance [3,4].

In this regard, several studies have demonstrated that inhibition of cyclooxygenase-2 (COX-2) significantly affects MDR1 (P-gp) regulation across different tumor models [5,6,7]. COX-2 catalyzes the production of prostaglandin E_2_ (PGE_2_), a bioactive molecule that acts as a ligand for G protein–coupled receptors and regulates key processes such as inflammation, cell survival, and chemoresistance [8]. At the molecular level, it has been proposed that increased PGE_2_ promotes activation of the JNK/c-Jun pathway, favoring the binding of this transcription factor to the MDR1 promoter and, consequently, its overexpression [5,9,10]. Additionally, COX-2 expression is tightly controlled by NF-κB, a transcription factor highly responsive to cellular redox status and widely implicated in inflammation-driven tumor progression [11,12]. Therefore, COX-2 emerges as a relevant therapeutic target, as inhibiting it could help reverse drug resistance [5,13].

Glioblastoma (GB) represents a paradigmatic model of therapeutic resistance, with a median survival of approximately 15 months post-diagnosis and a 5-year survival rate below 7%, despite current treatments [14]. Standard management includes surgical resection followed by radiotherapy and chemotherapy with temozolomide (TMZ), which has led to only a marginal improvement in overall survival, albeit with high inter-patient variability [15]. This scenario highlights the need to identify new agents that improve therapeutic response, particularly those capable of modulating inflammation-associated and redox-sensitive pathways.

Natural antioxidants derived from dietary sources have gained increasing attention as modulators of inflammation and cancer-related signaling. Among them, anthocyanins such as glycosylated delphinidins are widely distributed in berries and are recognized for their bioactive properties [16,17,18]. Notably, Chile represents a unique and strategic source of delphinidins, as it is a major producer of berries with exceptionally high anthocyanin content [19]. Among them, *Aristotelia chilensis*, a native and endemic plant of Chile whose fruit is known as maqui, stands out as one of the richest natural sources of glycosylated delphinidins [20]. This phytochemical richness supports the exploration of these compounds as bioactive agents in cancer research [21,22,23]. More recently, growing evidence has highlighted the potential of delphinidin-derived compounds as modulators of cancer-associated signaling pathways involved in drug resistance, oxidative stress, and inflammation. These compounds have been shown to regulate NF-κB, MAPK, PI3K/Akt, and ABC transporter-associated pathways across various tumor models, supporting their potential as adjuvant agents to enhance treatment response and overcome chemoresistance [23,24,25]. Our previous work showed that delphinidin-3-glucoside (DEL-3) enhances the anti-tumor effect of TMZ in GB models by reducing cell viability, potentially via inhibition of NF-κB signaling, supporting its role as a promising adjuvant [18]. Importantly, NF-κB has been described as a transcriptional regulator of COX-2, and there is abundant evidence indicating that delphinidins can modulate PGE_2_ production [26,27]. Taken together, this evidence suggests that glycosylated delphinidins may regulate MDR1 expression through an NF-κB/COX-2–dependent axis, although this mechanism has not been fully elucidated.

It should be noted that ABC transporters, particularly MDR1, play a critical physiological role at the blood–brain barrier (BBB) [28]. MDR1 is highly expressed in brain endothelial cells, where it actively exports xenobiotics and potentially harmful compounds from the central nervous system, thereby contributing to brain homeostasis and protection [29]. However, this same mechanism represents a major obstacle for brain cancer therapy, as MDR1 limits the penetration and retention of numerous anticancer agents within the brain parenchyma. In glioblastoma, MDR1 overexpression has been reported not only in BBB-associated endothelial cells but also in tumor cells, further reducing intracellular drug accumulation and contributing to treatment failure and chemoresistance [30]. Therefore, identifying molecules that can modulate their expression or function represents a promising strategy to improve glioblastoma treatment.

Among the glycosylated delphinidins present in anthocyanin-rich berries such as maqui, DEL-3 and delphinidin-3,5-diglucoside (DEL-3,5) are naturally occurring delphinidin glycosides that have attracted increasing interest because of their biological activities [20]. Although both compounds share the same delphinidin aglycone, they differ in the number and position of glycosyl residues, a structural feature known to influence stability, cellular uptake, bioavailability, and biological activity [31]. Therefore, DEL-3,5 was included in selected experiments as a structurally related comparator to explore whether differences in glycosylation could influence the regulation of MDR1 expression and associated signaling pathways in glioblastoma cells. Subsequently, we evaluated the effect of delphinidin-3-glucoside on MDR1 regulation and its impact on the sensitivity of glioblastoma cells to chemotherapeutic agents that are substrates of this transporter, to explore its potential as a modulator of chemoresistance.

## 2. Materials and Methods

### 2.1. Cell Culture

U87-MG cells (ATCC^®^ HTB-14) were obtained from ATCC^®^ (Manassas, VA, USA) and cultured in DMEM/F12 medium (Gibco, Thermo Fisher Scientific, Cat. No. 125000096, Waltham, MA, USA), supplemented with 10% fetal bovine serum (FBS; Gibco, Thermo Fisher Scientific, Cat. No. 12657-059, Waltham, MA, USA) and 1× antibiotic–antimycotic solution (100 U/mL penicillin, 100 µg/mL streptomycin and 25 µg/mL amphotericin B; Thermo Fisher Scientific, Waltham, MA, USA).

Patient-derived mesenchymal glioblastoma cells, designated as GBM38 (provided by Universidad Francisco de Vitoria, Madrid, Spain), were maintained in M21 medium prepared according to the method described [32], supplemented with 10% FBS (Gibco, Thermo Fisher Scientific, Cat. No. 12657-029, Waltham, MA, USA) and 1× antibiotic–antimycotic solution (Thermo Fisher Scientific, Waltham, MA, USA).

All cells were cultured in a humidified CO_2_ incubator (Air-Jacketed Automatic CO_2_ Incubator, Nuaire, Cat. No. UN-5100E, Plymouth, MN, USA) at 37 °C under 5% CO_2_ until reaching approximately 80% confluence. For subculturing, U87-MG cells were treated with trypsin (Gibco, Thermo Fisher Scientific, Cat. No. 25200-072, Waltham, MA, USA), while GBM38 cells were detached using accutase (Gibco, Thermo Fisher Scientific, Cat. No. 00-4555-56, Waltham, MA, USA) for 7–10 min at 37 °C and subsequently maintained in their respective culture media. Periodically, cells were routinely screened for mycoplasma contamination by PCR.

### 2.2. Cell Viability Assay

**Figure 1:**
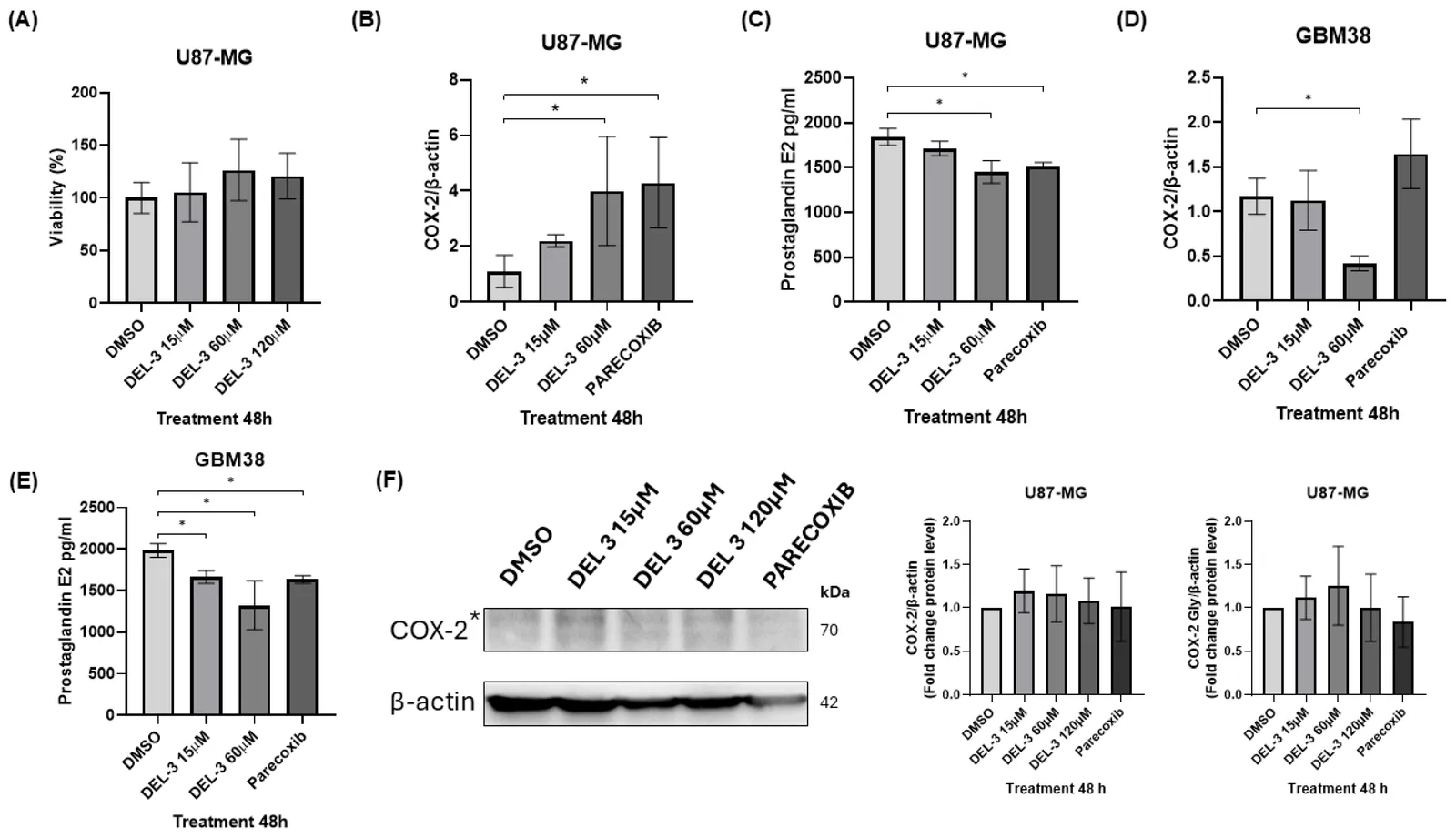
Reduction in PGE_2_ production and modulation of the COX-2 pathway in glioblastoma cells. (**A**) Cell viability in U87-MG cells after 48 h of treatment with delphinidin-3-glucoside (DEL-3). (**B**) COX-2 transcript levels in U87-MG cells treated with DEL-3 (15 and 60 μM) and parecoxib (20 μM) for 48 h. (**C**) PGE_2_ production in U87-MG cells under the same treatments, measured by ELISA. (**D**) COX-2 transcript levels in GBM38 glioblastoma stem-like cells treated with DEL-3 (15 and 60 μM) and parecoxib (20 μM) for 48 h. (**E**) PGE_2_ production in GBM38 cells under the same treatments, showing a marked reduction at both concentrations of DEL-3. (**F**) Western blot analysis of COX-2 protein levels (* Glycosylated form of COX-2), densitometric quantification of the non-glycosylated (~70 kDa) and glycosylated (~75 kDa) COX-2* bands is shown below. Data are presented as mean ± standard deviation (SD); *p* < 0.05. N = 3.

To evaluate cell viability, 96-well plates were seeded with U87-MG cells at a density of 5000 cells/well. Following a 24 h stabilization period, cells were treated with 120 µM delphinidin-3-glucoside (PHL89627, PhytoProof^®^, Merck, Darmstadt, Germany). This compound was administered either independently or alongside specific chemotherapeutic agents and inhibitors: 20 µM parecoxib (COX-2 inhibitor; Pro-Bextra, Pfizer), 0.1 µM vincristine, 0.5 µM doxorubicin, 1 µM etoposide (all three from Tocris Bioscience, Bristol, UK), or 0.5 µM paclitaxel (Sandoz, Basel, Switzerland). Post-treatment incubation was maintained for 24 and 48 h before performing the MTT assay. Briefly, 20 µL of a 5 mg/mL MTT stock solution (dissolved in culture medium) was introduced into each well. Cells were returned to 37 °C for 3 h to promote the accumulation of intracellular formazan crystals. The resulting crystals were subsequently solubilized by adding 100 µL of DMSO per well. After brief plate agitation, optical density was quantified at 570 nm using a BioTek Instruments microplate reader (Winooski, VT, USA). The concentrations of DEL-3 and DEL-3,5 (15, 60, and 120 μM) (PhytoProof^®^, Merck, Darmstadt, Germany) were selected based on our previous findings [18] and confirmed by the dose–response viability analysis shown in Figure 1A. Among these concentrations, 120 μM produced the greatest modulation of MDR1 expression while preserving sufficient cell viability and was therefore selected for subsequent mechanistic and combination studies.

### 2.3. Drug Combination and Synergy Analysis

To evaluate the nature of the interaction between delphinidin-3-glucoside and the selected chemotherapeutic agents or inhibitors, cell viability data obtained from fixed-dose combination experiments were analyzed using the SynergyFinder+ web application (https://synergyfinder.org/ accessed on 3 August 2026) [33,34]. Due to the single-point combination screening design where independent monotherapies were compared against fixed-dose pairs (e.g., 120 µM DEL-3 combined with specific single doses of 0.1 µM vincristine, 0.5 µM doxorubicin, 1 µM etoposide, or 0.5 µM paclitaxel), synergy calculations were restricted to individual cross-points rather than a full dose–response matrix. Drug interaction scores were calculated using the Bliss Independence reference model, which assumes that the compounds act through independent biological pathways. Synergistic effects were quantified via the Bliss score, where values greater than 10 indicated synergy, scores between −10 and 10 represented additive effects, and scores below −10 indicated antagonism. To complement the interaction analysis with phenotypic potency, the Combination Sensitivity Score (CSS) was determined for the specific single-point combinations to reflect the localized treatment efficacy on U87-MG cell viability.

### 2.4. Luciferase Reporter Vectors

To evaluate MDR1 promoter activity, the pmir-GLO/proMDR1 construct was used, based on the pmir-GLO Dual-Luciferase reporter vector (Promega, Cat. No. E1330, Madison, WI, USA). This vector was engineered by inserting an approximately 1200 bp fragment (GenBank: AY910577.1; positions 113,218–114,417) corresponding to the MDR1 promoter region upstream of the firefly luciferase (LUC2) coding sequence. Construct design was carried out using ApE (A Plasmid Editor; v3.1.7, M. Wayne Davis), and the final plasmid was synthesized and obtained from GenScript Biotech (Piscataway, NJ, USA).

Transient transfection of the pmir-GLO/proMDR1 vector was performed using TurboFect transfection reagent (Thermo Fisher Scientific), following the manufacturer’s instructions. Twenty-four hours post-transfection, cells were subjected to the corresponding experimental treatments.

### 2.5. RNA Extraction, Reverse Transcription, and RT-qPCR

Total RNA isolation from glioblastoma cells treated with glycosylated delphinidins was achieved utilizing TRIzol reagent (Invitrogen, Cat. No. 15596018, Carlsbad, CA, USA) according to the manufacturer’s protocol. Purification was subsequently carried out through standard chloroform–isopropanol extraction followed by 70% ethanol washing steps. Purified RNA was dissolved in nuclease-free water and preserved at −80 °C. For complementary DNA (cDNA) synthesis, a two-step reaction protocol was implemented to achieve a final volume of 20 µL. Initially, a mixture comprising 1 µg of total RNA, dNTPs, and 25 ng/mL Oligo(dT)15 primers was prepared in nuclease-free water, incubated at 65 °C for 5 min, and promptly chilled to 4 °C for 5 min. Next, a second master mix containing 1 U RNase inhibitor, 10 U Moloney murine leukemia virus reverse transcriptase (M-MLV RT), and 5× M-MLV buffer was incorporated. cDNA synthesis was completed by incubating the combined mixtures at 37 °C for 1 h (all synthesis reagents were sourced from Promega, Madison, WI, USA). Real-time PCR assays were conducted on a LineGene 9640 real-time PCR detection system (Bioer, Cat. No. 4334, Hangzhou, Zhejiang, China). The thermocycling profile included an initial denaturation phase at 95 °C for 10 min, followed by 40 amplification cycles consisting of denaturation at 95 °C for 15 s, annealing at 55 °C for 20 s, and extension at 72 °C for 20 s. To verify amplicon specificity, a melting curve analysis was performed by heating from 70 °C to 90 °C in 0.5 °C increments. PCR mixtures (15 µL final volume) were prepared with Brilliant II SYBR^®^ Green QPCR Master Mix (Agilent Technologies, Santa Clara, CA, USA), 1 µL of cDNA template, nuclease-free water, and 0.5 µM of forward and reverse primers (Table 1; Integrated DNA Technologies, Coralville, IA, USA). Endogenous expression of beta-actin was used as the reference control for normalization. Relative mRNA expression variations were quantified via the 2^−ΔΔCt^ method.

### 2.6. Western Blot

Whole-cell protein lysates were obtained using 200 µL of 1× RIPA lysis buffer (Invitrogen, Cat. No. 89000, Carlsbad, CA, USA) supplemented with 1× protease and phosphatase inhibitors (Roche, Basel, Switzerland). After adding the lysis solution to the pre-treated plates, cells were detached by scraping, and the resulting lysates were transferred to 1.5 mL Eppendorf tubes. Centrifugation was carried out at 14,000 rpm for 15 min at 4 °C using a high-speed SCILOGEX D3024R centrifuge (Rocky Hill, CT, USA). The collected supernatant was stored at −80 °C. Quantification of protein concentration was conducted with the Pierce BCA Protein Assay Kit (Pierce, Cat. No. 23225, Rockford, IL, USA) against a bovine serum albumin (BSA) standard curve (ranging from 2 to 0.125 mg/mL). In a 96-well plate, 10 µL of either standards or protein samples were mixed with 200 µL of the working reagent per well. Following a 30 min incubation at 37 °C, absorbance values were gathered at 595 nm on a Synergy 2 microplate reader (BioTek, Cat. No. 18531, Winooski, VT, USA).

Protein samples 20 µg were resolved via 12% SDS-PAGE using a Mini-PROTEAN II electrophoresis apparatus (Bio-Rad, Hercules, CA, USA). Prior to loading, samples were denatured at 95 °C for 5 min in a 6× loading buffer composed of 0.8% SDS, 240 mM Tris-HCl (pH 6.8), 40% glycerol, 200 mM beta-mercaptoethanol, and 0.02% bromophenol blue. Electrophoresis was performed at 80–100 V in a running buffer containing 0.1% SDS, 25 mM Tris, and 192 mM glycine (PanReac AppliChem, Barcelona, Spain). Resolved proteins were transferred onto nitrocellulose (Bio-Rad, Cat. No. 1620112) or PVDF membranes (Amersham Hybond, GE Healthcare, Amersham, Buckinghamshire, UK) utilizing a Bio-Rad semi-dry transfer system. The transfer buffer consisted of 20% methanol, 20 mM Tris, and 150 mM glycine (pH 8.3). Membrane blocking was sustained for 1 h at room temperature using a blocking buffer composed of TBS-T (0.5% Tween-20 in Tris-buffered saline, pH 7.6) supplemented with 5% BSA. Membranes were subsequently incubated overnight under gentle agitation at 4 °C with the corresponding primary antibodies. The next day, after washing the membranes three times with TBS-T, the membranes were incubated with appropriate secondary antibodies for 1 h at room temperature. Following three additional TBS-T washes, chemiluminescent signals were developed using SuperSignal™ West Dura substrate (Thermo Scientific, Rockford, IL, USA) and captured via a SYNGENE G: BOX imaging system (Synoptics Ltd., Cambridge, UK). Primary and secondary antibodies utilized in these analyses are detailed in Table 2, all diluted in TBS-T containing 0.005% Tween-20.

### 2.7. Luciferase Activity Assay

Transfection of U87-MG cells with purified reporter constructs was performed at 70–80% confluence in 24-well plates. Each well received a mixture of 500 ng of luciferase reporter plasmid and 2 µL of Lipofectamine 2000 (Invitrogen, Cat. No. 11668019, Carlsbad, CA, USA). During transfection, cells were maintained in serum-free Opti-MEM; this transfection medium was then replaced with complete culture medium after a 24 h incubation at 37 °C. Following a 24- or 48 h treatment window with glycosylated delphinidins, cells were lysed by adding 70 µL of passive lysis buffer to each well. Quantification of luciferase activity was performed using the Dual-Luciferase^®^ Reporter Assay System (Promega, Cat. No. E1910, Madison, WI, USA) according to the manufacturer’s protocol.

### 2.8. ELISA Assay

Bulleted A total of 50,000 GB cells (U87-MG or GBM38) were seeded per well in 24-well plates and incubated for 24 h to allow cell attachment. Cells were then treated with glycosylated delphinidins (DEL-3) at concentrations of 15 μM and 60 μM, using DMSO as a negative control and parecoxib as a positive control, for 48 h under standard culture conditions (37 °C, 5% CO_2_).

Following treatment, culture supernatants were collected and centrifuged at 1200 rpm for 10 min to remove cells and debris. The clarified supernatants were used to quantify prostaglandin E2 (PGE2) levels using a Prostaglandin E2 Parameter Assay Kit (Bio-Techne, Cat. No. KGE004B, Minneapolis, MN, USA), according to the manufacturer’s instructions.

Samples and standards were incubated in pre-coated antibody plates, and detection was performed using a colorimetric reaction measured at 405 nm using a Synergy 2 microplate reader (BioTek, Cat. No. 18531, Winooski, VT, USA).

### 2.9. Animal Model

Male NOD-SCID mice (Mus musculus), 6–8 weeks of age and weighing 20–35 g, were obtained from the Central Animal Facility of the Faculty of Medicine, Universidad de Chile. Animals were maintained under standard housing conditions and had no previous experimental procedures prior to inclusion in the study. A xenograft model was established in NOD-SCID mice via subcutaneous injection of 1 × 10^6^ U87-MG cells suspended in 100 μL of PBS. Beginning on day 7 post-inoculation, animals were treated via intraperitoneal injection every 72 h for 12 days (four doses), with an injection volume of 300 μL per dose.

Experimental groups included a control group treated with DMSO (1:10 in saline solution) and a treatment group receiving DEL-3 (10 mg/kg). The dose of 10 mg/kg DEL-3 was selected based on previous studies evaluating delphinidins in vivo [35]. A total of eight NOD-SCID mice were used in the study. Animals were allocated into two experimental groups: a vehicle-treated control group (*n* = 4) and a delphinidin-3-glucoside-treated group (*n* = 4). Sample size was determined based on previous xenograft studies and the statistical justification described in the approved animal protocol. The minimum number of animals required to obtain valid experimental results while adhering to the principles of Reduction, Replacement, and Refinement (3Rs) was used. Treatment administration was performed under blinded conditions. Experimental groups were identified using coded group numbers, and the investigator administering the treatments was unaware of group allocation. Tumor growth was monitored weekly by palpation and caliper measurements, and tumor volume was calculated using the formula:

V = (4/3)π × (length/2) × (width/2)^2^

Animals were followed for up to 3 weeks or until tumors reached a volume of 1200 mm^3^. At this endpoint, or at 4 weeks post-inoculation, mice were anesthetized with ketamine/xylazine and euthanized by cervical dislocation. Tumors were subsequently excised and stored at −80 °C for further analyses. No animals or data points were excluded from the analysis. All mice completed the study or reached the predefined humane endpoint (tumor volume ≥ 1200 mm^3^) and were included in the final analyses. The primary outcome measures were tumor volume and MDR1 expression in xenograft tissues. All animal procedures were conducted in accordance with institutional and national guidelines and were approved by the Institutional Committee for the Care and Use of Animals (CICUA) of the Universidad de Chile (protocol CBA 1218 FMUCH). Ethical approval was granted on 26 January 2024.

### 2.10. Immunohistochemistry

Formalin-fixed, paraffin-embedded tissue sections (4 μm) were mounted on positively charged slides, deparaffinized in xylene, and rehydrated through graded ethanol to distilled water. Antigen retrieval was performed by heating in citrate buffer (10 mM, pH 6.0). Endogenous peroxidase activity was blocked with 3% hydrogen peroxide for 10 min at room temperature, followed by incubation in blocking solution to reduce nonspecific binding. Sections were then incubated overnight at 4 °C with the primary antibody Anti-Mdr-1 (Dilution 1:200, Santa Cruz Biotechnology, Inc., Dallas, TX, USA). After washing with phosphate-buffered saline (PBS). Immunoreactivity was visualized using ImmPRESS^TM^ Excel Amplified HRP (peroxidase) Polymer Staining Kit (Vector Laboratories, Newark, CA, USA), according to the manufacturer’s instructions, and nuclei were counterstained with Mayer’s hematoxylin. Negative controls were processed in parallel by omitting the primary antibody. A blinded observer scored MDR1 immunoreactivity using a semi-quantitative scale (1 = low, 2 = moderate, 3 = high staining intensity). For visualization purposes, scores were converted to relative values (35%, 75%, and 100%) and plotted to compare experimental groups.

### 2.11. Statistical Analysis

Statistical analyses were performed using GraphPad Prism 8 (GraphPad Software, Inc., San Diego, CA, USA). Differences between two groups were evaluated using the Mann–Whitney test, while comparisons among multiple groups were conducted using one-way ANOVA followed by Tukey’s post hoc test. Data distribution was evaluated using the Shapiro–Wilk normality test prior to statistical analysis.

All experiments were performed with at least three biological replicates. Differences were considered statistically significant at *p* < 0.05.

## 3. Results

### 3.1. DEL-3 Reduces PGE_2_ Production and Modulates the COX-2 Pathway in Glioblastoma Cells

Glycosylated delphinidins have previously been reported to modulate prostaglandin production through the regulation of COX-2 activity. Based on this, the effect of DEL-3 on PGE_2_ production was evaluated as a functional readout of COX-2 activity in glioblastoma cells. U87-MG and GBM38 cells were treated with DEL-3 at concentrations of 15 and 60 μM for 48 h, using parecoxib (20 μM) as a positive control for COX-2 inhibition. First, cell viability analysis showed that DEL-3 treatment did not significantly affect U87-MG cell viability at the tested concentrations, indicating that the observed effects were not attributable to cytotoxicity (Figure 1A).

The expression of COX-2 transcripts in U87-MG cells was assessed after 48 h of treatment. A significant increase in COX-2 mRNA levels was observed at 60 μM DEL-3, whereas the 15 μM concentration did not induce notable changes. Interestingly, parecoxib treatment also led to a moderate elevation in COX-2 transcripts, suggesting a potential compensatory response to reduced PGE_2_ production (Figure 1B). Interestingly, COX-2 functional output, as measured by PGE_2_ production, was significantly reduced in U87-MG cells at 60 μM DEL-3, despite the observed increase in COX-2 expression (Figure 1C). In contrast, the 15 μM concentration did not produce significant changes. In GBM38 cells, DEL-3 induced a significant decrease in COX-2 mRNA expression, particularly at 60 μM, indicating transcriptional downregulation (Figure 1D). Parecoxib treatment resulted in a slight, non-significant increase in COX-2 transcripts. Consistent with these findings, DEL-3 markedly reduced PGE_2_ production at both tested concentrations (15 and 60 μM), suggesting that GBM38 cells are more sensitive to DEL-3 treatment compared to U87-MG cells (Figure 1E). The magnitude of this effect was comparable to that observed with parecoxib.

Western blot analysis in U87-MG cells showed that DEL-3 treatment did not significantly modify total COX-2 protein levels at any of the concentrations tested (15, 60, or 120 μM), despite the transcriptional changes observed and the reduction in PGE_2_ production (Figure 1F). COX-2 was detected as a doublet between ~70 and 75 kDa, consistent with distinct glycosylation states, with the upper band (~75 kDa) corresponding to the glycosylated form. Separate densitometric analyses of the non-glycosylated and glycosylated forms revealed no statistically significant differences between treatment groups, although a trend toward increased COX-2 protein abundance was observed following DEL-3 exposure.

These findings indicate that the marked reduction in PGE_2_ production induced by DEL-3 cannot be explained by changes in total COX-2 protein abundance or its glycosylated form. This dissociation between COX-2 protein levels and enzymatic output suggests that DEL-3 may regulate the COX-2/PGE_2_ axis through mechanisms that affect enzyme activity or downstream components of prostaglandin biosynthesis, rather than by modulating COX-2 protein expression itself. Moreover, the differential response observed between U87-MG and GBM38 cells underscores the importance of cellular context. GBM38 cells, which display glioblastoma stem-like properties, exhibit a distinct molecular response to COX-2 modulation. Taken together, these results indicate that DEL-3 reduces PGE_2_ production while promoting a cell-type–dependent regulation of COX-2 expression at both transcriptional and protein levels. To further investigate the apparent dissociation between COX-2 expression and PGE_2_ production in U87-MG cells, PTGES1 (microsomal prostaglandin E synthase-1), the terminal enzyme responsible for PGE_2_ synthesis, was assessed by RT-qPCR in U87-MG cells after 48 h of treatment (Figure S1). DEL-3 significantly increased PTGES1 transcription levels to 120 μM, whereas BAY11-7082, parecoxib, and the JNK inhibitor did not induce comparable changes. These findings indicate that the reduction in PGE_2_ production observed after DEL-3 treatment cannot be explained by transcriptional repression of PTGES1 and instead suggest the activation of compensatory regulatory mechanisms within the prostaglandin synthesis pathway.

### 3.2. MDR1 Expression Is Reduced in the Presence of Glycosylated Delphinidins in Glioblastoma Cells

ABCB1 transcript levels were evaluated by RT-qPCR in U87-MG cells treated with glycosylated delphinidins (DEL-3 and DEL-3,5) for 24 and 48 h, using parecoxib as a positive control for COX-2 pathway inhibition and DMSO as a negative control.

**Figure 2:**
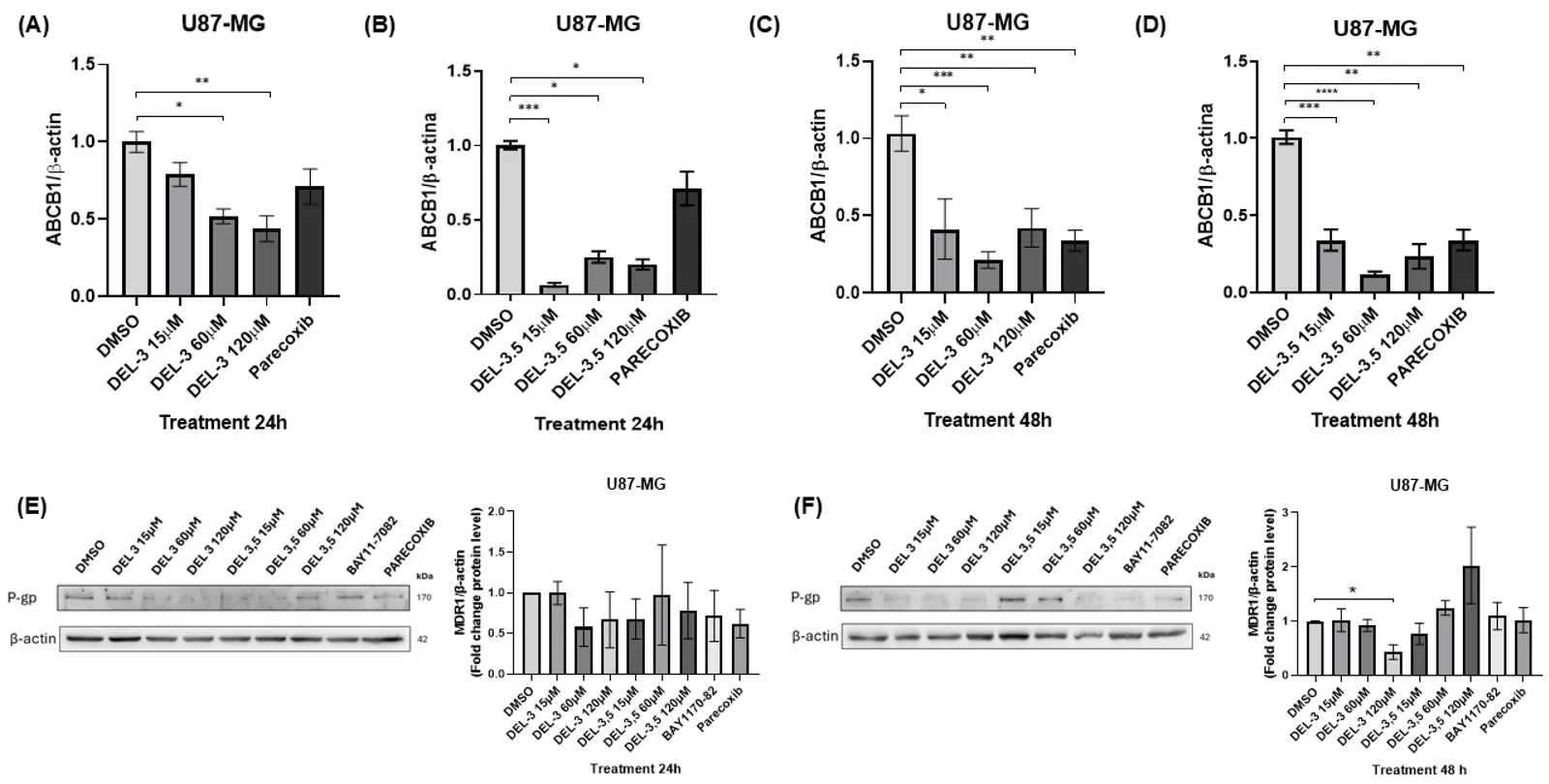
MDR1 transcript and protein levels in U87-MG cells treated with glycosylated delphinidins for 24 and 48 h. (**A**) ABCB1 transcript expression in U87-MG cells treated for 24 h with DEL-3 at 15, 60, and 120 μM, with parecoxib (20 μM) as a positive control. (**B**) ABCB1 transcript expression in U87-MG cells treated for 24 h with DEL-3,5 under the same conditions. (**C**) ABCB1 transcript expression in U87-MG cells treated for 48 h with DEL-3. (**D**) ABCB1 transcript expression in U87-MG cells treated for 48 h with DEL-3,5. (**E**) P-gp (MDR1) protein levels determined by Western blot in U87-MG cells treated for 24 h with DEL-3 or DEL-3,5; an NF-κB inhibitor (BAY11-7082, 10 nM) and parecoxib (20 μM) were used as controls; the graph on the right shows the densitometric quantification of the P-gp (MDR1). (**F**) P-gp protein levels determined by Western blot in U87-MG cells treated for 48 h with DEL-3 or DEL-3,5; the same controls were used as described above; the graph on the right shows the densitometric quantification of the P-gp (MDR1). Data are presented as mean ± standard deviation (SD); * *p* < 0.05; ** *p* < 0.01; *** *p* < 0.001; **** *p* < 0.0001. N = 3.

At 24 h, DEL-3 treatment significantly reduced ABCB1 transcript levels at concentrations of 60 and 120 μM, indicating a dose-dependent effect. Notably, the inhibition observed at 120 μM was more pronounced than that induced by parecoxib (20 μM), which showed a weaker effect under these conditions (Figure 2A).

At 48 h, DEL-3 exposure resulted in a significant decrease in ABCB1 expression at all tested concentrations (15, 60, and 120 μM), indicating a dose-independent effect comparable to that observed with parecoxib (Figure 2C). These results suggest that DEL-3 exerts a sustained inhibitory effect on MDR1 at the transcriptional level.

In contrast, delphinidin-3,5-diglucoside (DEL-3,5) treatment showed a distinct pattern. At 24 h, a significant reduction in ABCB1 levels was observed at all concentrations, with the strongest effect detected at the lowest dose (15 μM), suggesting that maximum inhibition was already achieved at the lowest concentration tested (Figure 2B). At 48 h, this downregulation was maintained across all conditions, again without a clear dose-dependent pattern (Figure 2D).

At the protein level, MDR1 (P-gp) expression was evaluated by Western blot analysis. Parecoxib and BAY11-7082, an inhibitor of the NF-κB pathway, were included as mechanistic controls.

At 24 h, densitometric analysis did not reveal significant changes in MDR1 protein abundance following treatment with either DEL-3 or DEL-3,5 (Figure 2E), despite the reductions observed at the transcript level.

At 48 h, DEL-3 treatment induced a significant reduction in MDR1 protein levels only at the highest concentration tested (120 μM), whereas lower concentrations did not produce significant changes (Figure 2F). In contrast, DEL-3,5 failed to significantly decrease MDR1 protein expression at any concentration. Likewise, BAY11-7082 and parecoxib did not significantly affect MDR1 protein levels under these experimental conditions.

Taken together, these findings indicate that the transcriptional downregulation of MDR1 induced by DEL-3 is only partially reflected at the protein level, with a significant reduction detected exclusively after 48 h of treatment at 120 μM. These results support the notion that DEL-3 can modulate MDR1 expression in glioblastoma cells, although additional post-transcriptional and post-translational regulatory mechanisms may influence the final abundance of MDR1 protein.

### 3.3. Delphinidin-3-Glucoside Regulates MDR1 Promoter Activity in Glioblastoma Cells

To evaluate the effect of glycosylated delphinidins on the transcriptional regulation of MDR1 (ABCB1), promoter activity was analyzed in U87-MG cells transfected with the dual-luciferase reporter vector pmir-GLO/proMDR1, using Renilla luciferase activity as an internal control. Cells were treated with DEL-3 or DEL-3,5 (15, 60, and 120 μM) for 24 and 48 h, including parecoxib as a positive control for COX-2 pathway inhibition.

**Figure 3:**
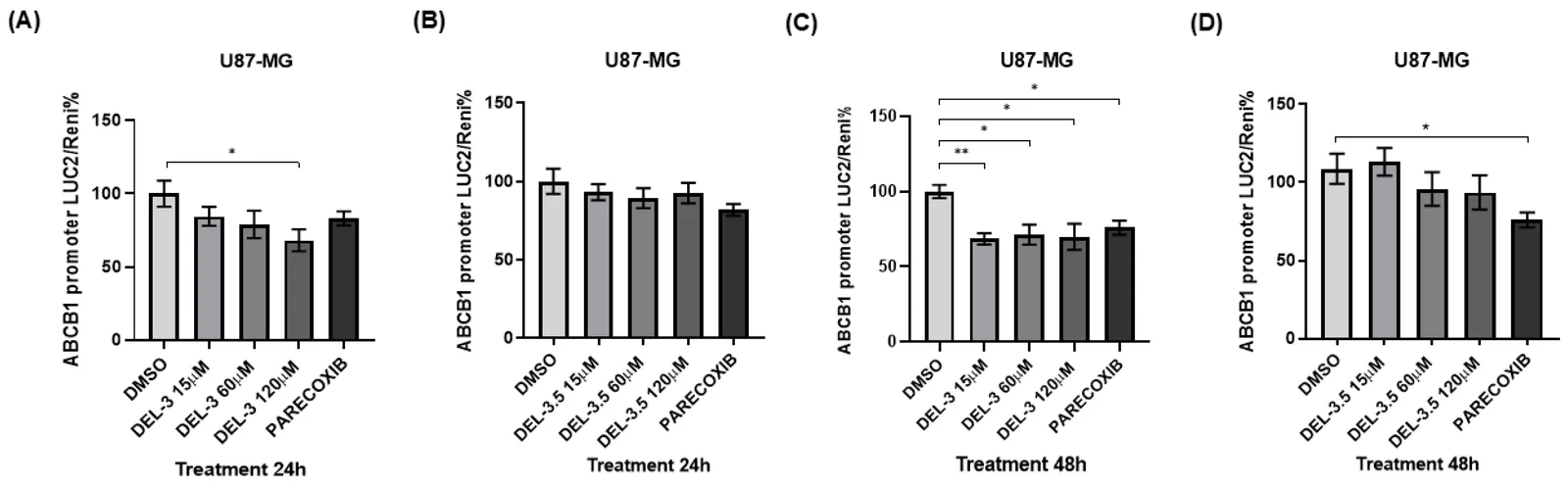
ABCB1 promoter activity in glioblastoma cells treated with glycosylated delphinidins. (**A**) ABCB1/MDR1 promoter activity in U87-MG cells transfected with the pmir-GLO/proMDR1 reporter vector and treated for 24 h with DEL-3 at 15, 60, and 120 μM (**B**) MDR1 promoter activity in U87-MG cells treated for 24 h with DEL-3,5 at the same concentrations. (**C**) MDR1 promoter activity in U87-MG cells treated for 48 h with DEL-3. (**D**) MDR1 promoter activity in U87-MG cells treated for 48 h with DEL-3, 5. In all cases, parecoxib (20 μM) was used as a positive control to inhibit the COX-2 pathway. Data are presented as mean ± standard deviation (SD); * *p* < 0.05; ** *p* < 0.01. N = 3.

At 24 h, DEL-3 treatment induced a significant decrease in MDR1 promoter activity only at the 120 μM concentration, whereas lower concentrations showed no significant effects (Figure 3A). This effect was more pronounced than that observed with parecoxib, which did not reach statistical significance under these conditions. In contrast, DEL-3,5 treatment did not produce detectable changes in promoter activity at any of the tested concentrations (Figure 3B).

At 48 h, DEL-3 treatment was associated with a significant reduction in MDR1 promoter activity at all tested concentrations, indicating a dose-independent effect (Figure 3C). The magnitude of inhibition was comparable to that observed with parecoxib, which also suppressed promoter activity. In contrast, DEL-3,5 continued to show no significant effect on promoter activity even after prolonged exposure (Figure 3D).

Taken together, these results indicate that DEL-3, but not DEL-3,5, negatively regulates MDR1 promoter activity in a time-dependent manner. These findings suggest that MDR1 inhibition by DEL-3 occurs, at least in part, at the transcriptional level in glioblastoma cells.

### 3.4. Correlation Between ABCB1 Expression and Delphinidin-Regulated Genes in Glioblastoma Patients

To evaluate the clinical relevance of MDR1 regulation, the correlation between ABCB1 expression and genes previously reported to be modulated by delphinidins in cellular models was analyzed using transcriptomic data from 225 glioblastoma patients obtained from the Chinese Glioma Genome Atlas CGGA dataset (Gliovis).

**Figure 4:**
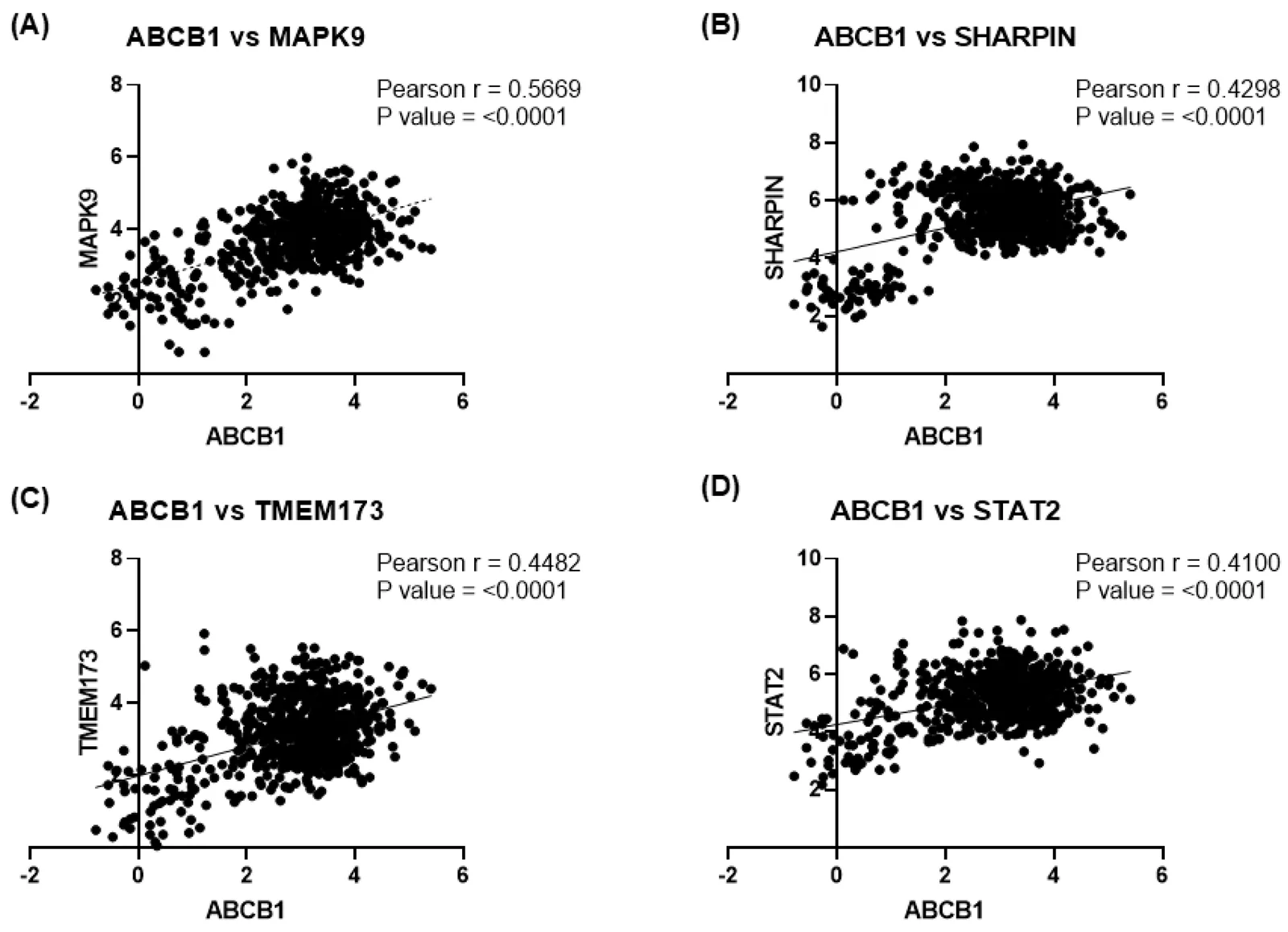
Correlation of ABCB1 expression levels with delphinidin-regulated markers in Gliovis data. Data from the CGGA dataset (Gliovis), including 225 glioblastoma cases, were analyzed using Pearson correlation. (**A**) Correlation between ABCB1 and MAPK9 expression levels. (**B**) Correlation between ABCB1 and SHARPIN. (**C**) Correlation between ABCB1 and TMEM173. (**D**) Correlation between ABCB1 and STAT2.

Pearson correlation analysis revealed a positive correlation between ABCB1 and MAPK9 (r = 0.566, *p* < 0.0001), suggesting a potential link with JNK signaling (Figure 4A). Similarly, a positive correlation was observed between ABCB1 and SHARPIN (r = 0.42, *p* < 0.0001), indicating possible co-regulation within pathways associated with inflammatory signaling and cell survival (Figure 4B).

Additionally, ABCB1 expression was positively correlated with TMEM173 (r = 0.44, *p* < 0.0001), a gene involved in STING pathway activation and innate immune responses (Figure 4C). Finally, a positive correlation was found between ABCB1 and STAT2 (r = 0.41, *p* < 0.0001), which may reflect an association with interferon-dependent signaling pathways and a potential connection with NF-κB signaling (Figure 4D).

Taken together, these positive correlations suggest that ABCB1/MDR1 expression is integrated into a transcriptional program involving genes previously shown to be regulated by delphinidins, consistent with our prior findings. These observations support the hypothesis that MDR1 expression may be associated with inflammatory and immune signaling networks relevant to glioblastoma biology. However, these correlations do not demonstrate that DEL-3 directly regulates these pathways in patients.

### 3.5. MDR1 Levels Are Reduced in the Presence of DEL-3 in Glioblastoma Xenografts

**Figure 5:**
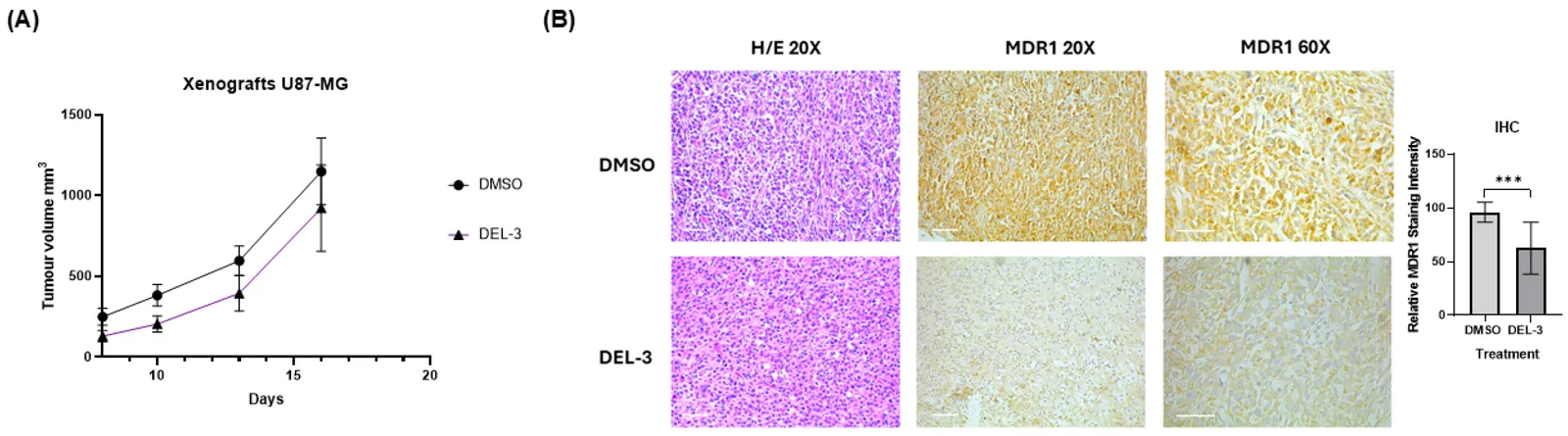
MDR1 levels in glioblastoma xenografts treated with delphinidin-3-glucoside. (**A**) Tumor volumes were assessed in NOD-SCID mice injected with U87-MG cells and treated with DEL-3 up to day 16 post-treatment. (**B**) Representative histological sections of xenografts stained with hematoxylin and eosin (H/E), along with IHC staining for MDR1 at 20× and 60× magnification. Relative MDR1 staining intensity was quantified and is shown in the graph. The scale bar represents 50 μm. Representative of N = 4. *** *p* < 0.001.

Tumor growth analysis in the NOD-SCID xenograft model showed no significant differences in tumor volume between animals treated with delphinidin-3-glucoside and those in the DMSO control group. In both groups, tumors exhibited progressive growth, reaching comparable sizes up to day 16 post-treatment, at which point animals were euthanized according to the established experimental criteria (Figure 5A).

Tumors were subsequently excised, fixed, and embedded in paraffin for histological analysis. Evaluation of MDR1 protein levels by immunohistochemistry (IHC) revealed that xenografts derived from DEL-3–treated animals displayed reduced MDR1 expression compared to tumors from the DMSO-treated control group (Figure 5B).

Taken together, these results indicate that although DEL-3 treatment does not significantly affect tumor growth in this in vivo model, it does modulate MDR1 expression. This downregulation demonstrates in vivo modulation of the target. However, because DEL-3 was administered as a single agent and no chemotherapy combination group was included, the capacity of DEL-3 to enhance chemosensitivity in vivo remains to be established.

### 3.6. Delphinidin-3-Glucoside Enhances the Cytotoxic Effects of MDR Substrate Drugs in Glioblastoma Cells

To evaluate the potential of delphinidin-3-glucoside (DEL-3) as a chemosensitizing agent, cell viability was assessed in U87-MG cells following treatment with different MDR1 substrate drugs, either alone or in combination with DEL-3 or parecoxib.

**Figure 6:**
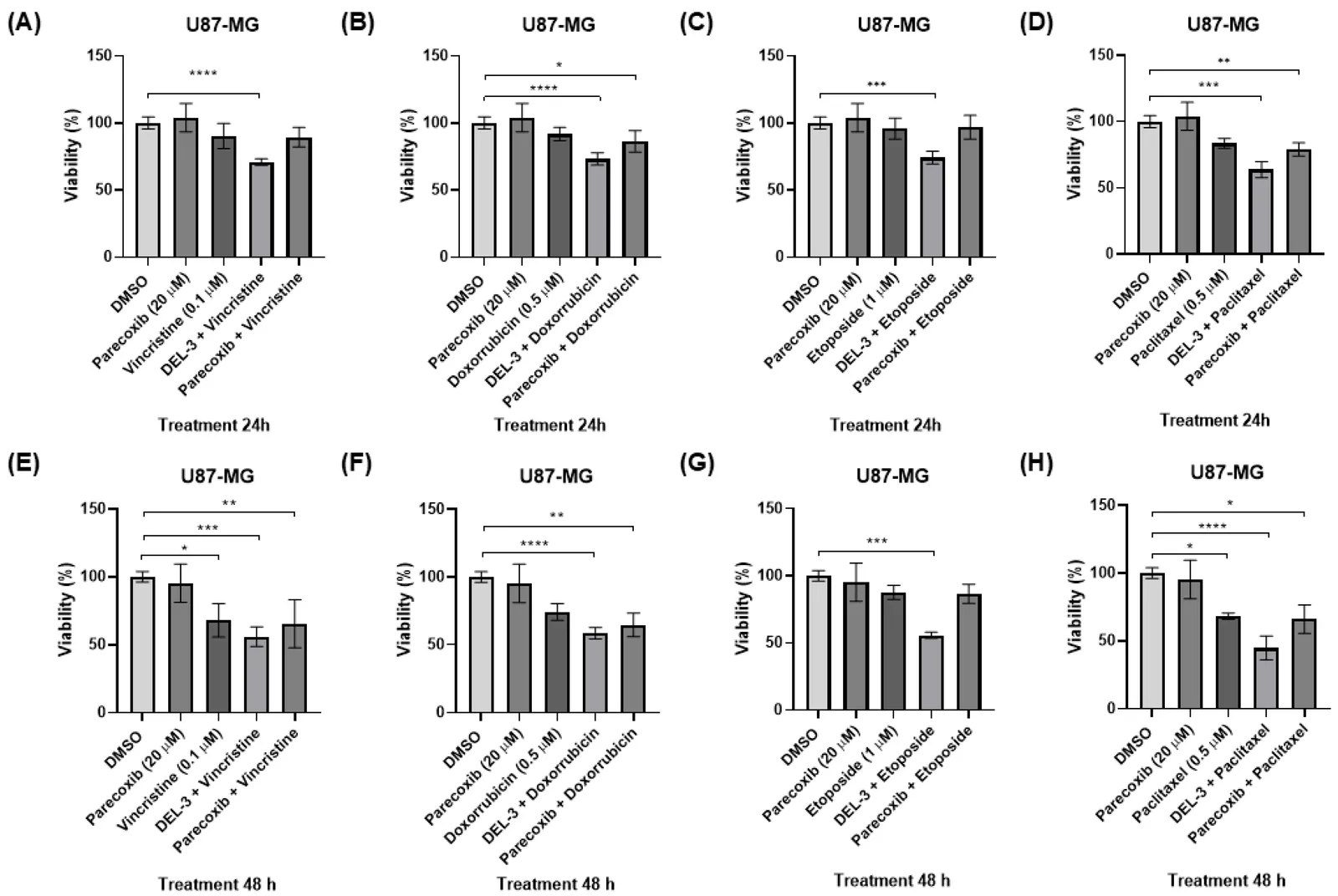
Effect of delphinidin-3-glucoside on chemosensitization of U87-MG glioblastoma cells. Cell viability was evaluated after treatment with MDR1 substrate chemotherapeutic agents in combination with DEL-3. (**A**) Cells treated with vincristine (0.1 μM), parecoxib (20 μM), DEL-3 (120 μM), and their combinations, showing a marked reduction in viability with the vincristine + DEL-3 combination. (**B**) Cells treated with doxorubicin (0.5 μM) under the same conditions. (**C**) Cells treated with etoposide (0.5 μM) in combination with DEL-3 and controls. (**D**) Cells treated with paclitaxel (0.5 μM) in combination with DEL-3 and controls. (**E**–**H**) Equivalent assays performed in U87-MG cells after 48 h of exposure to vincristine, doxorubicin, etoposide, or paclitaxel, administered alone or in combination with DEL-3 or parecoxib. Data are presented as mean ± standard deviation (SD); * *p* < 0.05; ** *p* < 0.01; *** *p* < 0.001; **** *p* < 0.0001. N = 3.

At 24 h, vincristine (0.1 μM) did not induce significant changes in cell viability. However, co-treatment with DEL-3 at 120 μM reduced viability (Figure 6A). Similarly, doxorubicin treatment alone for 24 h did not produce significant effects; however, its combination with DEL-3 (120 μM) or parecoxib led to a significant decrease in cell viability, with a more pronounced effect observed in combination with DEL-3 (Figure 6B).

For etoposide (1 μM), no changes in viability were observed when administered alone for 24 h. In contrast, co-treatment with DEL-3 significantly reduced cell viability (Figure 6C). Likewise, paclitaxel (0.5 μM) did not significantly affect cell viability when used as a single agent but showed a significant reduction when combined with DEL-3 (Figure 6D).

After 48 h of treatment, the effects on cell viability were more pronounced, particularly under co-treatment conditions with DEL-3. In this context, vincristine alone significantly reduced viability, and this effect was further enhanced when combined with DEL-3. Although co-treatment with parecoxib also decreased viability, the effect was comparable to that of vincristine alone (Figure 6E).

In the case of doxorubicin, significant reductions in cell viability at 48 h were only observed under combination conditions with either DEL-3 or parecoxib, again highlighting the stronger effect of DEL-3 (Figure 6F). For etoposide, a marked decrease in viability was observed exclusively when combined with DEL-3 (Figure 6G).

Finally, paclitaxel showed a significant cytotoxic effect after 48 h when administered alone, and this effect was further enhanced in the presence of DEL-3. In contrast, co-treatment with parecoxib resulted in a similar effect to paclitaxel alone (Figure 6H).

Taken together, these results indicate that DEL-3 consistently enhances the cytotoxic effects of multiple MDR1-substrate chemotherapeutic agents in vitro, suggesting its potential as a chemosensitizing agent in U87-MG glioblastoma cells.

Drug interaction landscapes and treatment potencies for the combination of DEL-3 120 µM with frontline chemotherapeutic agents were evaluated at 24 and 48 h using the SynergyFinder+ pipeline. All metric values calculated through the Bliss reference model and the CSS are summarized in Table S1 (24 h) and Table S2 (48 h). At 24 h, all tested combinations presented positive Bliss synergy scores above the standard threshold of 10. The highest interaction values in this interval were observed for combinations with etoposide (Bliss: 34.93) and doxorubicin (Bliss: 30.55), which were correlated with CSS values of 21.86 and 17.64, respectively. Combinations with vincristine and paclitaxel also showed synergy scores above 10, with initial CSS values of 17.49 and 13.74, respectively. At 48 h, distinct temporal changes were recorded across the treatments. While the synergy scores for vincristine, doxorubicin, and paclitaxel stabilized or slightly decreased compared with the 24 h assessment, their corresponding CSS values increased to 33.54, 37.11, and 29.58, respectively. In contrast, the combination with etoposide showed an increase in both evaluation metrics over time, reporting a Bliss score of 44.16 and a CSS of 40.88 at the 48 h interval.

## 4. Discussion

Our results showed that treatment with DEL-3 for 48 h in U87-MG cells did not significantly affect cell viability at concentrations of 15, 60, and 120 μM. This finding is consistent with previous work from our group, in which no differences in viability were observed at the same concentrations after 72 h of exposure [18].

The results obtained in this study suggest that glycosylated delphinidins, particularly DEL-3, act as modulators of the COX-2/PGE_2_ axis by significantly reducing PGE_2_ production in glioblastoma models [23]. This observation aligns with previous reports demonstrating that polyphenols can interfere with inflammatory signaling and limit prostaglandin synthesis [36,37,38]. Furthermore, our findings that DEL-3 reduces PGE_2_ production in glioblastoma cells are consistent with studies in other models, in which extracts enriched in delphinidin-3-O-glucoside decreased COX-2 and TNF-α expression and induced autophagy [36]. Collectively, these findings reinforce the role of delphinidins as modulators of inflammation and highlight their potential impact on MDR1 regulation. Notably, our data reveal a dissociation between COX-2 expression and functional output. While DEL-3 modulated COX-2 mRNA and protein levels in a cell-dependent manner, PGE_2_ production was consistently reduced. This suggests that DEL-3 does not simply regulate COX-2 expression but rather affects the functional output of the pathway. Similar observations have been reported for COX-2 inhibitors, in which reduced prostaglandin production can trigger compensatory upregulation of COX-2 expression through feedback mechanisms [39].

In this context, the reduction in PGE_2_ observed with DEL-3 treatment may result from functional inhibition of the COX-2 pathway at the enzymatic or post-enzymatic level, rather than from suppression of COX-2 expression. Potential mechanisms may include modulation of COX-2 catalytic activity, alteration of redox-dependent regulation, or interference with downstream enzymes such as microsomal prostaglandin E synthase-1 (mPGES-1), ultimately leading to reduced PGE_2_ synthesis despite preserved or increased COX-2 levels [40,41]. Interestingly, DEL-3 increased PTGES1 transcript expression despite reducing PGE_2_ production, suggesting the activation of compensatory regulatory mechanisms within the prostaglandin synthesis pathway. However, because neither COX-2 nor mPGES-1 enzymatic activities were directly evaluated, the specific molecular target responsible for the reduction in PGE_2_ production cannot be identified from the current data. This limitation should be addressed in future studies by directly assessing enzyme activity and prostaglandin biosynthetic flux. The reduction in PGE_2_ observed with DEL-3 treatment remains comparable to that achieved with the pharmacological inhibitor parecoxib, supporting the idea that DEL-3 functionally impacts the COX-2/PGE_2_ signaling axis. These findings further suggest that delphinidins may influence the regulation of genes linked to this pathway.

DEL-3 treatment induced a dose-dependent downregulation of MDR1 transcript levels, achieving a more pronounced effect than the COX-2 inhibitor parecoxib, suggesting a robust transcriptional mechanism of action. In contrast, DEL-3,5 also reduced MDR1 transcript expression, particularly at lower concentrations, but without a clear dose-dependent pattern, suggesting that glycosylation may influence the mechanisms through which these compounds regulate MDR1 expression. At the protein level, however, the effects of both compounds were less evident than those observed at the transcript level. Densitometric analysis revealed that DEL-3 significantly reduced MDR1 protein expression only after 48 h of treatment at 120 μM, whereas lower concentrations did not produce statistically significant effects. Likewise, despite its marked inhibitory effect on MDR1 transcripts, DEL-3,5 failed to significantly decrease MDR1 protein abundance under the conditions evaluated. This apparent disconnect between mRNA and protein levels suggests the involvement of additional post-transcriptional and/or post-translational regulatory mechanisms governing MDR1 expression in glioblastoma cells.

Notably, our data demonstrates that DEL-3 negatively regulates MDR1 promoter activity in glioblastoma cells in a time-dependent manner: at 24 h only at high concentrations (120 μM), and at 48 h in a sustained, dose-independent manner. These findings are consistent with reports describing other COX-2 modulators, such as celecoxib, which downregulate MDR1 expression in hepatocarcinoma via inhibition of PGE_2_ production and AP-1–mediated signaling [42]. Similarly, studies in breast cancer have shown that DEL-3-glucoside can regulate critical transcriptional pathways, such as the Akt/HOTAIR axis, influencing the expression of genes involved in tumor progression [43].

Taken together, these findings indicate that DEL-3 exerts a more sustained regulatory effect on MDR1, with transcriptional repression being partially translated into reduced protein abundance at higher concentrations and longer exposure times. By contrast, the effects of DEL-3,5 appear to be primarily restricted to transcript regulation and were not reflected at the protein level, highlighting potential mechanistic differences between these structurally related glycosylated delphinidins.

Figure 4 presents Pearson correlation analyses based on markers previously identified in our earlier study [18], derived from protein array data in U87-MG cells treated with DEL-3 or DEL-3,5. These correlations provide insights into potential functional links between the observed molecular changes and MDR1 regulation. Notably, a strong correlation was identified with MAPK9 (JNK2), which was upregulated by DEL-3,5. This is particularly relevant, as previous studies have demonstrated that the JNK pathway can modulate MDR1 expression, either through AP-1 binding to its promoter or through inhibition of YB-1 nuclear translocation, a key regulator of MDR1 transcription [44].

In this context, the lack of changes in MDR1 promoter activity following DEL-3,5 treatment may be partially explained by its dual regulatory effects, including JNK activation and NF-κB inhibition—both key signaling pathways involved in MDR1 regulation [18]. Additionally, positive correlations between ABCB1, TMEM173, and SHARPIN suggest that MDR1 expression may be associated with inflammatory signaling networks relevant to glioblastoma biology. However, these transcriptomic correlations do not demonstrate that DEL-3 directly regulates these pathways in patients. The correlation with STAT2, which is also downregulated by DEL-3, suggests an additional link to immune and interferon-related signaling pathways.

Figure 5 shows that, although DEL-3 treatment did not significantly alter tumor growth in NOD-SCID xenografts, it led to a clear reduction in MDR1 expression as determined by immunohistochemistry. This observation indicates that DEL-3 modulated a molecular target associated with chemoresistance in vivo. However, because DEL-3 was evaluated as a single agent and no chemotherapy-combination groups were included in the xenograft study, the impact of this reduction on therapeutic response remains unknown. These findings therefore support target modulation rather than direct evidence of in vivo chemosensitization. Previous reports have indicated that glycosylated anthocyanins, including DEL-3, exert minimal direct effects on MDR1 transporter activity, showing only weak ATPase stimulation and no significant inhibition in intestinal or blood–brain barrier transport models [45]. Our findings in glioblastoma cells indicate that DEL-3 consistently reduced MDR1 transcript expression and, under prolonged exposure, was also associated with reduced MDR1 protein abundance, particularly at higher concentrations. In vitro, this reduction correlated with increased sensitivity to several MDR1-substrate chemotherapeutic agents (Figure 6), supporting a potential role for DEL-3 in functionally modulating chemoresistance mechanisms. Several limitations of this study should be acknowledged. First, the xenograft experiments evaluated DEL-3 as a single agent and therefore do not directly demonstrate chemosensitization in vivo. Second, pharmacokinetic parameters, blood–brain barrier penetration, and intratumoral exposure of DEL-3 were not assessed. Third, the NOD-SCID model lacks a functional adaptive immune system and therefore cannot fully recapitulate immune-inflammatory interactions that may be involved in NF-κB/COX-2 signaling. Additionally, although DEL-3 enhanced the cytotoxic effects of several MDR1-substrate chemotherapeutic agents, the specific mechanisms of cell death responsible for this response were not investigated. Future studies incorporating flow cytometry-based apoptosis and cell-cycle analyses, as well as complementary cell death assays, will be necessary to determine the molecular basis of the observed chemosensitizing effects. Finally, the proposed mechanistic links between NF-κB, JNK, COX-2/PGE_2_ signaling, and MDR1 regulation are based on associative evidence and should be validated through dedicated gain- and loss-of-function studies. Future studies should validate the effects of DEL-3 in additional patient-derived models representing distinct molecular subtypes and therapeutic resistance profiles. An additional consideration concerns the potential interactions between delphinidin-derived compounds and drug-metabolizing enzymes. Several polyphenols have been reported to modulate cytochrome P450 activity, including CYP3A4, which is involved in the metabolism of numerous anticancer agents [46]. Although such interactions were not evaluated in the present study, they should be considered when assessing the translational potential of DEL-3 as a chemotherapy adjuvant. Consequently, future pharmacokinetic and toxicological studies will be required to evaluate possible drug–drug interactions and their impact on treatment safety.

**Figure 7:**
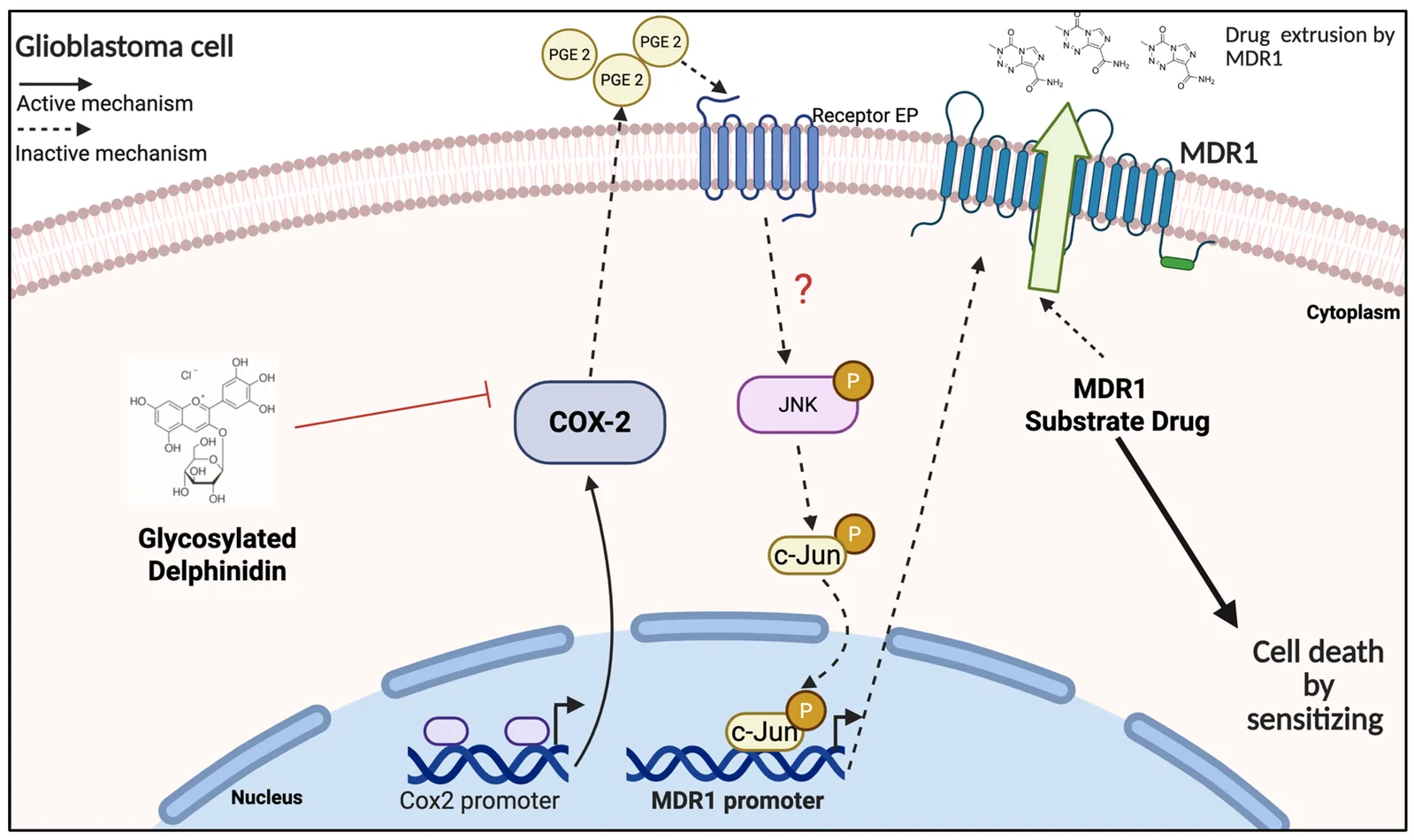
Proposed functional model in which delphinidin-3-glucoside (DEL-3) modulates the COX-2/PGE_2_ axis and reduces PGE_2_ levels, leading to downregulation of MDR1 expression. In vitro, this functional modulation was associated with increased sensitivity of glioblastoma cells to MDR1-substrate chemotherapeutic agents. Created in BioRender. Munoz, J. (2026) https://BioRender.com/2rxzpk3. Accessed on 3 August 2026.

In conclusion, while these findings do not constitute definitive proof, they provide a strong basis for proposing functional hypotheses regarding the differential effects of DEL-3 on MDR1 regulation. These hypotheses should be further explored in future studies to clarify the underlying molecular pathways. Finally, a schematic model summarizing the main findings of this study is presented (Figure 7).

## Figures and Tables

**Table 1:** Primer sequences used in RT-qPCR.

Target Gene	Accession Number	Forward (5′-3′)	Reverse (5′-3′)
*MDR1*	NM_000927	CCCAGGAGCCCATCCTGT	CCCGGCTGTCTCCATA
*β-actin*	NM_001101	GAGCACAGAGCCTCGCCTTT	CACGATGGAGGGGAAGACG
*PTGES1*	NM_004878.5	CTTCCTTTTCCTGGGCTTCG	GAAGACCAGGAAGTGCATCCA

**Table 2:** Antibodies used in Western blotting.

Antibody	Dilution	Company	Código
Anti-β-actin (C4): sc-47778 HRP	1:5000	Santa Cruz Biotechnology, Inc., Dallas, TX, EE.UU.	SC47778
Anti-Mdr-1 (D-11): sc-55510	1:500	Santa Cruz Biotechnology, Inc., Dallas, TX, EE.UU.	SC55510
Anti-COX-2 (H3): sc-376861	1:500	Santa Cruz Biotechnology, Inc., Dallas, TX, EE.UU.	
Goat anti-mouse IgG-HRP: 115-035-166	1:5000	Jackson ImmunoResearch, West Grove, PA, EE. UU	111-035-14
Goat anti-rabbit IgG-HR: 111-035-144	1:1000	Jackson ImmunoResearch, West Grove, PA, EE. UU	115-035-00

## Data Availability

The datasets generated and analyzed during the current study are publicly available in the Zenodo repository (DOI: 10.5281/zenodo.21814881). The repository contains the complete experimental dataset supporting the findings of this study. The PowerPoint file includes the original GraphPad Prism objects, allowing direct access to the underlying raw data by opening the presentation in Microsoft PowerPoint and double-clicking the corresponding graphs.

## Supplementary Materials

The following supporting information can be downloaded at: https://www.mdpi.com/article/10.3390/antiox15081005/s1. Figure S1; Relative PTGES1 mRNA expression in U87-MG glioblastoma cells following treatment with delphinidin-3-glucoside (DEL-3). U87-MG cells were treated with DEL-3 (15, 60, or 120 μM), BAY 11-7082, Parecoxib (20 μM), or the JNK inhibitor SC202671 (0.5 μM) for 48 h. PTGES1 transcript levels were determined by RT-qPCR and normalized to normalized to β-actin using the 2^−ΔΔCt^ method. Data are presented as mean ± SD from three independent experiments (*n* = 3). * *p* < 0.05. Table S1; Bliss synergy scores and Combination Sensitivity Scores (CSS) obtained from fixed-dose combination experiments using delphinidin-3-glucoside (DEL-3) together with vincristine, doxorubicin, etoposide, or paclitaxel in U87-MG glioblastoma cells after 24 h of treatment. Drug interaction analyses were performed using the SynergyFinder+ platform and the Bliss Independence reference model. Bliss scores > 10 indicate synergistic interactions, values between −10 and 10 indicate additive effects, and values < −10 indicate antagonism. CSS values represent the overall efficacy of each drug combination at the evaluated dose combination. Table S2; Bliss synergy scores and Combination Sensitivity Scores (CSS) obtained from fixed-dose combination experiments using delphinidin-3-glucoside (DEL-3) together with vincristine, doxorubicin, etoposide, or paclitaxel in U87-MG glioblastoma cells after 48 h of treatment. Drug interaction analyses were performed using the SynergyFinder+ platform and the Bliss Independence reference model. Bliss scores > 10 indicate synergistic interactions, values between −10 and 10 indicate additive effects, and values < −10 indicate antagonism. CSS values represent the overall efficacy of each drug combination at the evaluated dose combination.

## Abbreviations

The following abbreviations are used in this manuscript:

ABCB1	ATP-binding cassette subfamily B member 1
ABC	ATP-binding cassette
AP-1	Activator protein 1
BSA	Bovine serum albumin
cDNA	Complementary DNA
COX-2	Cyclooxygenase-2
DEL-3	Delphinidin-3-glucoside
DEL-3,5	Delphinidin-3,5-diglucoside
DMSO	Dimethyl sulfoxide
ELISA	Enzyme-linked immunosorbent assay
FBS	Fetal bovine serum
GB	Glioblastoma
IHC	Immunohistochemistry
JNK	c-Jun N-terminal kinase
LUC2	Firefly luciferase gene
MDR	Multidrug resistance
MDR1	Multidrug resistance protein 1
miRNA	MicroRNA
MTT	3-(4,5-dimethylthiazol-2-yl)-2,5-diphenyltetrazolium bromide
NF-κB	Nuclear factor kappa B
PBS	Phosphate-buffered saline
PGE_2_	Prostaglandin E2
P-gp	P-glycoprotein
qPCR	Quantitative polymerase chain reaction
RIPA	Radioimmunoprecipitation assay buffer
RT-qPCR	Reverse transcription quantitative polymerase chain reaction
SDS-PAGE	Sodium dodecyl sulfate polyacrylamide gel electrophoresis
TBS-T	Tris-buffered saline with Tween-20
TMZ	Temozolomide
TNF-α	Tumor necrosis factor alpha
