# Supplementary material for: Glycosylated Delphinidins, Natural Antioxidants, Functionally Modulate the COX-2/PGE2 Axis and Downregulate MDR1 in Glioblastoma Cells
Source: Antioxidants (Basel). 2026 Aug 13;15(8):1005. doi: 10.3390/antiox15081005 (PMC13510136; doi:10.3390/antiox15081005)
Supplement: Supplementary file 1 [file antioxidants-15-01005-s001.zip › antioxidants-4387586-supplementary.pdf]

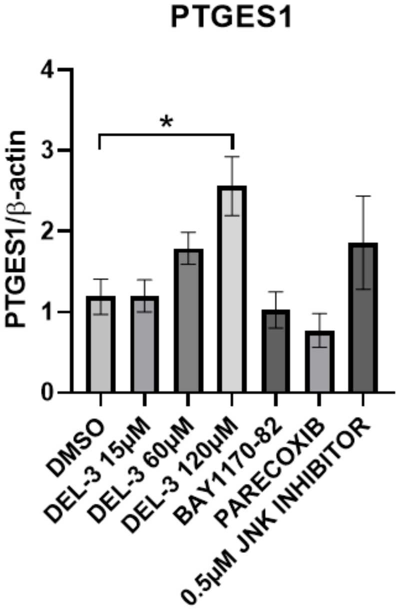

Figure S1. Relative PTGES1 mRNA expression in U87-MG glioblastoma cells following treatment with delphinidin-3-glucoside (DEL-3). U87-MG cells were treated with DEL-3 (15, 60, or 120 μM), BAY 11-7082, Parecoxib (20 μM), or the JNK inhibitor SC202671 (0.5 μM) for 48 h. PTGES1 transcript levels were determined by RT-qPCR and normalized to normalized to β-actin using the 2^−ΔΔCt method. Data are presented as mean ± SD from three independent experiments (n = 3). *p < 0.05.

Table S1. Bliss synergy scores and Combination Sensitivity Scores (CSS) obtained from fixed-dose combination experiments using delphinidin-3-glucoside (DEL-3) together with vincristine, doxorubicin, etoposide, or paclitaxel in U87-MG glioblastoma cells after 24 h of treatment . Drug interaction analyses were performed using the SynergyFinder+ platform and the Bliss Independence reference model. Bliss scores >10 indicate synergistic interactions, values between −10 and 10 indicate additive effects, and values <−10 indicate antagonism. CSS values represent the overall efficacy of each drug combination at the evaluated dose combination.

Drug Combination	Synergy Score Bliss	Sensitive Score CSS
DEL-3 - Vincristine	26.33	17.49
DEL-3 - Doxorubicin	30.55	17.64
DEL-3 - Etoposide	34.93	21.86
DEL-3 - Paclitaxel	18.68	13.74

Table S2. Bliss synergy scores and Combination Sensitivity Scores (CSS) obtained from fixed-dose combination experiments using delphinidin-3-glucoside (DEL-3) together with vincristine, doxorubicin, etoposide, or paclitaxel in U87-MG glioblastoma cells after 48 h of treatment. Drug interaction analyses were performed using the SynergyFinder+ platform and the Bliss Independence reference model. Bliss scores >10 indicate synergistic interactions, values between −10 and 10 indicate additive effects, and values <−10 indicate antagonism. CSS values represent the overall efficacy of each drug combination at the evaluated dose combination.

Drug Combination	Synergy Score Bliss	Sensitive Score CSS
DEL-3 - Vincristine	16.71	33.54
DEL-3 - Doxorubicin	25.30	37.11
DEL-3 - Etoposide	44.16	40.88
DEL-3 - Paclitaxel	17.62	29.58
